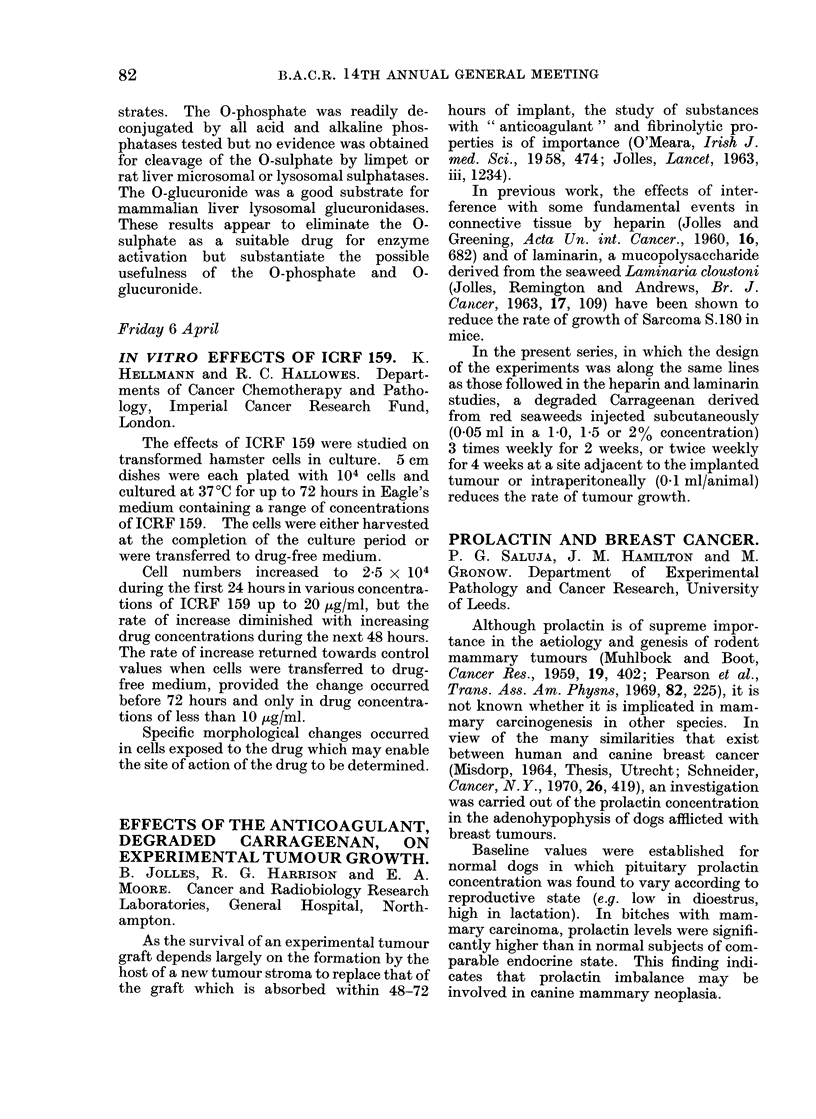# Prolactin and breast cancer.

**DOI:** 10.1038/bjc.1973.95

**Published:** 1973-07

**Authors:** P. G. Saluja, J. M. Hamilton, M. Gronow


**PROLACTIN AND BREAST CANCER.** P. G. SALUJA, J. M. HAMILTON and M. GRONOW. Department of Experimental Pathology and Cancer Research, University of Leeds.

Although prolactin is of supreme importance in the aetiology and genesis of rodent mammary tumours (Muhlbock and Boot, *Cancer Res.*, 1959, **19**, 402; Pearson *et al.*, *Trans. Ass. Am. Physns*, 1969, **82**, 225), it is not known whether it is implicated in mammary carcinogenesis in other species. In view of the many similarities that exist between human and canine breast cancer (Misdorp, 1964, Thesis, Utrecht; Schneider, *Cancer, N.Y.*, 1970, **26**, 419), an investigation was carried out of the prolactin concentration in the adenohypophysis of dogs afflicted with breast tumours.

Baseline values were established for normal dogs in which pituitary prolactin concentration was found to vary according to reproductive state (*e.g.* low in dioestrus, high in lactation). In bitches with mammary carcinoma, prolactin levels were significantly higher than in normal subjects of comparable endocrine state. This finding indicates that prolactin imbalance may be involved in canine mammary neoplasia.